# Fellow Perceptions of Program Culture Conveyed Through Virtual Interviews: Aligning Perceptions With Reality

**DOI:** 10.7759/cureus.62637

**Published:** 2024-06-18

**Authors:** Jieun David, Suhagi Kadakia, Beverley Robin

**Affiliations:** 1 Pediatrics, Rush University Children's Hospital, Chicago, USA; 2 Neonatology, Rush University Children's Hospital, Chicago, USA

**Keywords:** social media, culture, training program, medical education, virtual interview

## Abstract

Objective: Due to the COVID-19 pandemic, medical education training programs had to rapidly switch to a virtual interview (VI) format for the 2021 application cycle. Studies have demonstrated a gap in applicant perceptions of program culture through the VI. The objectives of this study were to assess the perceptions of culture from VIs compared to direct experience after beginning training and explore VI gaps in representing culture.

Study design: An anonymous questionnaire was emailed to first-year neonatal-perinatal medicine fellows who participated in the 2020-2021 and 2021-2022 VI process. Demographic and interview data and information regarding the presentation of and gaps in the portrayal of program culture through VIs were collected. Responses were evaluated using descriptive statistics, the Wilcoxon signed-rank test, and qualitative analysis.

Results: Eighty-five survey responses were received. In determining how well the respondent perceptions of program culture from the VI aligned with their direct experiences, respondent scores showed a median of 80 (scale of 0-100%) with an IQR of 57.5-90. There were significant differences in the perception of fellow-faculty relationships (p = 0.044), the priority placed on fellow teaching (p < 0.001), respect and value for fellows (p = 0.001), and fellow work-life integration (p = 0.004). Nineteen percent of respondents reported not meeting with fellows during their VI and only 15% reported usage of social media in their VI. Respondents noted fellows to be the most important people contributing to perceptions of program culture and provided possible solutions to address challenges in representing culture.

Conclusion: Despite the small number of respondents, the quantitative and qualitative results offer enlightening information on the gaps in presenting culture through VIs. Notably, the perception of program culture from the VI did not align well with direct experience, particularly in areas addressing fellow relationships and the value placed on fellow teaching, respect, and work-life integration. Increasing fellow involvement, arranging informal settings, and the usage of social media may be important tools to improve accuracy in the representation of culture through VIs.

## Introduction

The novel coronavirus (COVID-19) pandemic led to a rapid switch from in-person to virtual interview (VI) format for graduate and post-graduate medical education programs, including neonatal-perinatal medicine (NPM) programs in the 2021 application cycle. This required programs to adapt their interview process without precedent or guidance from the literature. Since then, studies have demonstrated the advantages of VIs, including financial savings for applicants and programs, reduced time away from clinical responsibilities, reduced carbon emissions, time efficiency, less pressure during interviews, the ability for applicants to interview at a greater number of programs and a more equitable interview process [1-7]. However, studies show that the lack of in-person connection makes it difficult for applicants to assess a program’s culture, as the perception of culture is influenced by direct and personal interactions [1,3,6,8-11]. To our knowledge, no studies have explored the gaps in the representation of program culture through VIs, especially from a multi-institutional perspective. We conducted this study to 1) compare first-year NPM fellows’ perceptions of the culture of the program into which they matched, based on the VI, to their direct experience of the program’s culture after beginning their training and 2) explore the VI format and activities used by NPM fellowship programs to identify gaps in representation of program culture.

## Materials and methods

Study design

This was a cross-sectional survey of first-year NPM fellows in the USA and Canada. The study was deemed exempt by the Institutional Review Board at Rush University Medical Center (22022501-IRB01). Survey completion served as consent for participation and responses were anonymous.

A 28-question online survey was developed by three board-certified neonatologists with experience in graduate and post-graduate medical education. The survey included twelve 5-point Likert questions directed toward the perception of program culture based on the VI (before matching into the program) and perception of program culture based on direct experience (after beginning training). One question asked participants to rate (0-100%) how well their perception of program culture from the VI aligned with their perception through direct experience. Six single/multiple answer questions related to the VI format and fellow/faculty attitudes toward applicants during the VIs. Two free-text questions explored what most significantly contributed to the perception of program culture during the VIs and what NPM fellows would like programs to include to convey program culture. Six demographic questions were included.

Survey distribution

In May 2022 and January 2023, the survey instructions and electronic survey link were distributed via email to all USA Accreditation Council for Graduate Medical Education (ACGME)-accredited NPM program directors through the Organization of Neonatal Training Program Directors (ONTPD) listserv and directly to all Canadian NPM program directors. Program directors were asked to distribute the survey instructions and link via e-mail to their current first-year NPM fellows who participated in the 2020-2021 and 2021-2022 VI process, respectively (for academic year 2021-2022 and 2022-2023). An additional request for survey distribution was sent to program directors in June 2022 and March 2023. Responses were collected via the web-based software REDCap (Research Electronic Data Capture v13.6.1). To ensure anonymity, a survey link that does not allow identification was utilized.

Data analysis

Data were sorted within REDCap with responses aggregated and analyzed using descriptive statistics, including proportions for categorical data. Respondents were exited from the survey if they had previous work or training experience at their training institution. Incomplete responses were excluded from the analysis. Likert scale responses were measured on a scale of 1-5 with 5 representing "strongly agree." Individual respondents' answers regarding their perception of program culture were compared based on their VI experience versus their direct experience. Changes in median value between perception of program culture based on VIs and perception of program culture based on direct experience were calculated and analyzed using a Wilcoxon signed-rank test to assess the significance of differences in perception (IBM SPSS Statistics for Windows, Version 27 (Released 2020; IBM Corp., Armonk, New York, United States)). Free text responses were analyzed with thematic mapping and collated with descriptive statistics.

## Results

Eighty-five surveys were received with 61 surveys included in data collection and reporting. Fifty (83.3%) respondents were from the USA and 10 (16.7%) were from Canada. The majority of respondents listed their program type as a university hospital, and most received their medical training from a US medical school. Table 1 shows respondent demographic data.

**Table 1:** Respondent demographics The data has been presented as N (%). Incomplete responses were excluded from analysis with proportions reflecting total responses received for each subcategory. MD: Doctorate of Medicine; DO: Doctorate of Osteopathic Medicine

	Respondents, N (%)
Age	
25-30	18 (30.0)
31-35	31 (51.7)
36-40	7 (11.7)
>40	4 (6.7)
Gender	
Female	37 (60.7)
Male	22 (36.1)
Prefer not to say/Other	2 (3.2)
Other	1 (1.6)
Credentials	
MD from US medical school	34 (55.7)
MD from non-US medical school	17 (27.9)
DO from US medical school	2 (3.3)
MBBS	4 (6.6)
Other	4 (6.6)
Country of fellowship training	
USA	50 (83.3)
USA: Northeast	21 (42.9)
USA: Southeast	5 (10.2)
USA: Midwest	13 (26.5)
USA: Southwest	3 (6.1)
USA: West	7 (14.3)
Canada	10 (16.7)
Program type	
University hospital	53 (86.9)
Community hospital, university-affiliated	7 (11.5)
Other	1 (1.6)

**Figure 1:**
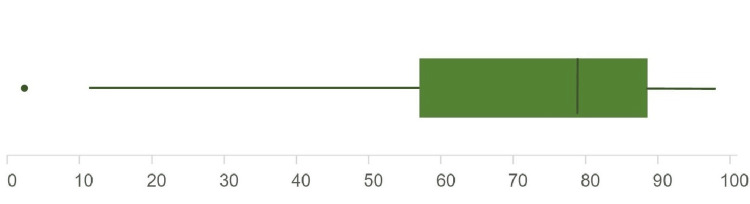
Respondents' ratings of accuracy in perception of culture through virtual interview versus direct experience Respondents' ratings of how accurately program culture as portrayed through virtual interviews aligns with their direct experience of program culture. Box plot representing median (IQR) of 80 (57.5,90), with 100 representing greater correlation.

Respondents’ ratings of how well their perception of program culture from VIs aligned with their direct experience showed a median of 80 (scale of 0-100, with higher value representing greater correlation) with an IQR of 57.5-90 (Figure 1).

**Figure 2:**
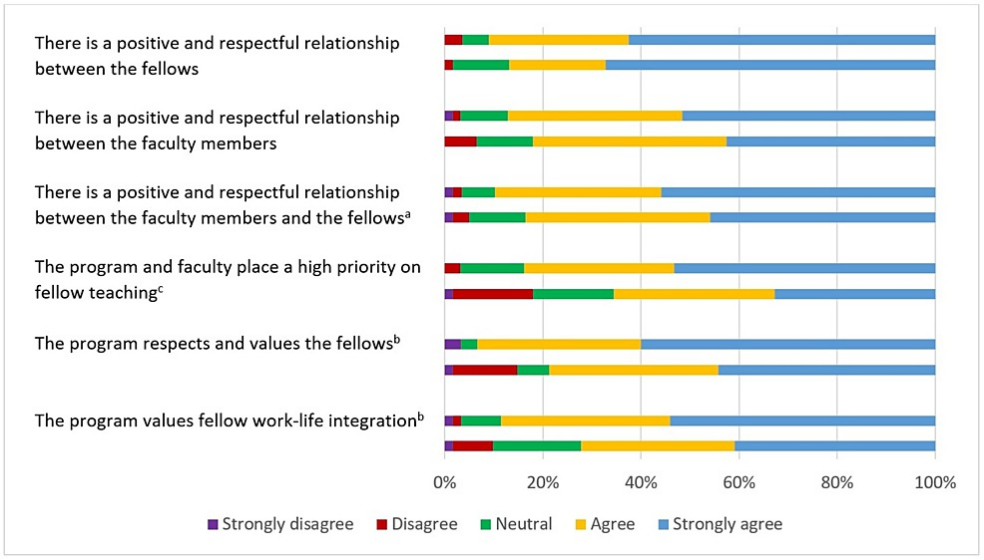
Respondent perceptions of program culture based on virtual interviews versus direct experience For each question, respondent perceptions of program culture are displayed, with the top row reflecting perceptions based on the virtual interview and bottom row reflecting perceptions based on direct experience after beginning training. ^a^p-value < 0.05; ^b^p-value < 0.01; ^c^p-value < 0.001

Figure 2 shows the paired analysis for respondent perceptions of program culture from the VI compared to direct experience. There were significant differences in the perception of fellow-faculty relationships (p = 0.044), priority placed on fellow teaching (p < 0.001), respect and value for fellows (p = 0.001), and fellow work-life integration (p = 0.004). Regarding attitudes toward applicants on the VI day, 53 (88%) respondents reported that fellows were very or somewhat positive, while 59 (97%) reported that faculty were very or somewhat positive.

**Figure 3:**
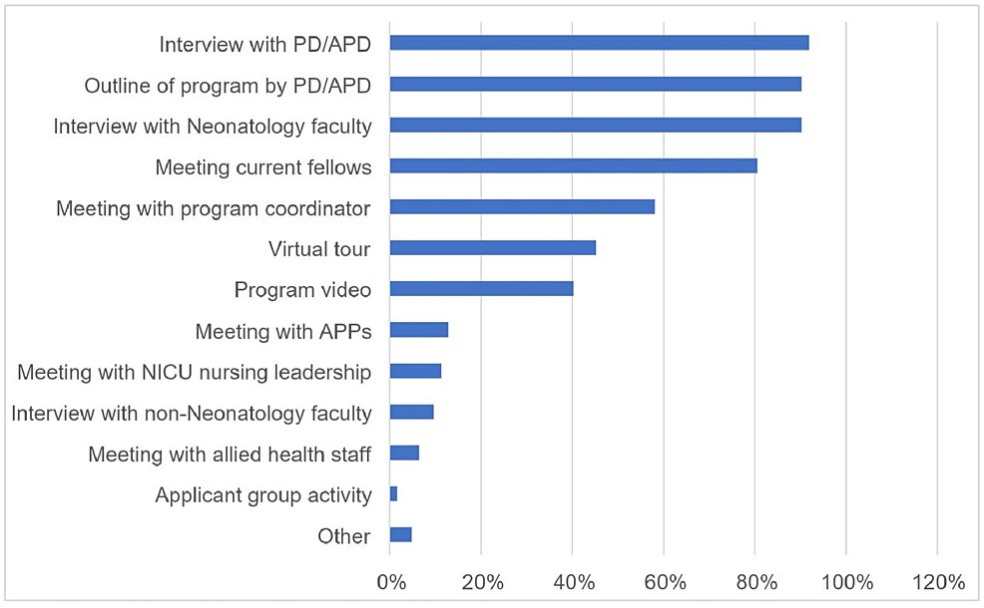
Activities included on interview day NICU: neonatal intensive care unit; APD: associate program director; PD: program director; APP: advanced practice provider

Most interview days were two to four hours (N=27, 43.5%) or five to six hours (29, 46.8%) in duration. Figure 3 shows the activities included in the interview day. Only 80.6% (N=50) of respondents met with the program’s current fellows during the VI day, and 23.0% (N=14) of programs gave applicants an opportunity to meet with the current fellows virtually at a time separate from the interview day. Eight (12.9%) respondents met with advanced practice providers (APPs) during their interview day and less than half of the programs (N=25, 40.3%) included a program video. Only nine (15%) respondents reported that programs used social media to provide information regarding their interview day or program activities and events.

Analysis of free text responses showed that the people who contributed most to the perception of program culture through the VI were the current fellows, program director, neonatology faculty, and program coordinator, respectively, with fellows mentioned 1.5 times more than any other person. Many respondents mentioned “interactions between fellows” as the largest contributor to their perception of program culture, with one respondent stating: “my meeting with the fellows embodied the program culture.” Recurrent themes were interactions, relationships, and conversations, with respondents describing both conversing with fellows and/or faculty and observing interactions between fellows and/or faculty as helpful in understanding program culture.

The VI activities that were most frequently mentioned as contributors to the perception of program culture included interviews, an introduction or description of the program, virtual happy hours, and question-and-answer sessions. Some respondents’ comments were: “[the] fellow and faculty happy hour was very enlightening” and “during the virtual happy hour with fellows, it was clear they enjoyed each other.” When asked about activities, respondents most frequently wished that the VI day included meeting with fellows and having longer, separate, or smaller group meetings. Example quotes were: “I would have welcomed the opportunity to meet with the fellows for a little longer” and [I wish my program’s VI had included] “informal discussions with the fellows.” Several respondents said they wished to meet with the fellows without coordinators or staff present. One commented: “I wish the fellows weren’t all in one room. That made it difficult to see and interact with them. I did a second look for this reason and just spoke to two fellows individually. It was a much better experience and [I] got a better feel for the fellows. We changed this the following year for interviews based on our feedback.”

Another recurrent theme was the lack of interviews or meetings with APPs or other staff, such as nursing leadership. One respondent said, “Meeting with APPs would have been nice. A significant portion of our day is working with them so that would have helped give a feel to the overall culture.” Another commented, “I wish I somehow could have seen fellow-APP dynamics during my interview day.” Regarding program description content, respondents frequently wished for virtual tours of the neonatal intensive care unit (NICU) and hospital, including tours of other rotating sites and videos of the program or the day-to-day life of a fellow. Example quotes included: “It would have been nice to see what the NICU unit would look like”; “I would have liked a virtual tour of the unit”; and [I wish my program’s VI had included] “a short video about the program objectives, staff, the unit [and] people that are parts of the team” and “information via social media.”

## Discussion

This novel study is the first to compare NPM fellows’ perceptions of program culture based on VIs with their direct experience of program culture after beginning training, and the first study to specifically explore the gaps in representation of culture in the VI format outside of the experience of a single institution. Though the number of respondents was small, the data provided is essential to understanding the gaps in the presentation of culture through VIs. Notably, we found that program culture represented via VIs did not align well with respondents’ direct experience of program culture. Our findings from the paired analysis showed a significant discrepancy between perceived program culture through VIs and direct experience of program culture, especially as it relates to the faculty and fellow relationship, the priority placed on fellow teaching, and the degree to which the program respects and values the fellows and work-life integration.

Several studies have similarly reported limitations in the general assessment of a program via the VI format, including difficulty in evaluating an applicant’s fit and having a good sense of program faculty or fellow-faculty relationships [2,8-10,12,13]. A survey of obstetrics and gynecology applicants and faculty by Armstrong et al. found that most applicants (77%) agreed or strongly agreed that they were able to develop connections during the VI, but less than half of applicants felt that VIs helped them assess their fit for a program [14]. In a study of general surgical oncology fellow applicants, Grova et al. found that those who attended in-person interviews believed they had an accurate perception of the program culture (100%), whereas only 64% of VI applicants had the same impression [15]. Despite the advantages of VI, studies consistently demonstrate an increased difficulty in assessing program culture.

Importantly, the paired analysis revealed multiple differences in perceptions of culture from the VI, particularly regarding the respect and value placed on fellows, fellow teaching, work-life integration, and fellow-faculty relationships. Applicant perspectives on these factors are often gathered through direct conversation or observed interactions with current fellows. Prior studies have reported current trainees as a primary resource used to create the most accurate impression of a program [12,16,17]. Similarly, in our study, we found that the fellows were most frequently reported to influence applicant perceptions of program culture compared to any other people. Despite this, almost 20% of survey respondents reported that they did not meet with fellows during their VI. Historically, current trainees have been a large part of the in-person NPM program interview process, typically with opportunities for dynamic and organic interactions; omitting their involvement in the interview process creates a notable gap in forming a candidate’s general perspective of a program and of the program culture.

Our study highlights the need to set up private and comfortable meetings between candidates and fellows to allow for natural interactions and honest feedback. Studies have shown that the lack of personal connection during VIs leads to difficulty in detecting nonverbal social cues, including eye contact and body language, and that applicants perceive VI conversations to be “forced” and “awkward” [7,18]. Allowing for smaller group interactions in a non-interview format may be beneficial in fostering a more casual environment and personal connections. Faculty behaviors are also affected by the formality of VIs; in Brian et al.’s study on VIs, faculty expressed that “[virtual] interviews are more about us selling our program” [9]. In our findings, faculty attitudes were perceived as being more positive compared to fellow attitudes, potentially relating to this underlying objective. In surveying urologic oncology fellowship applicants, Gore et al. found that the lack of informal interactions with fellows and faculty was a limitation of VIs [19]. Previous studies have supported having social events or other opportunities for informal interactions with fellows or faculty to improve gaps in understanding program culture [9,19,20]. Similarly, happy hour and Q&A sessions were mentioned by our survey respondents as helpful in assessing program culture. Czawlytko et al.’s study of radiology residency applicants reported that 91% of applicants attended their virtual dinner or happy hour events and 63% reported that a virtual dinner or happy hour influenced their program ranking [21]. Providing these external opportunities may present a less stressful environment for both applicants and program representatives and offer a different perspective of the training community.

Surprisingly, only 15% of survey respondents reported that programs used social media in the VI process to inform them about program activities or events. Social media is a powerful tool that can portray aspects of a program’s culture and community that might not be conveyed during the VI day. In their evaluation of social media in VIs, Czawlytko et al. found that almost half believed that social media played a vital role in the application process and 71% used social media to learn more about the program [21]. Similarly, a study of California medical students by Naaseh et al. showed that more than half followed prospective residency programs’ social media accounts and 89% were influenced by the programs’ social media [22]. Additionally, 86% believed that programs should use social media during future application cycles even if interviews are in-person, and Facebook and Instagram were the top two digital approaches used to learn about programs [22]. In addition to showcasing a program’s “brand,” social media is an effective way to present program information and show a trainee’s day-to-day life. Many programs have fellow spotlights and fellow events showing the interaction between fellows. These examples may reflect a program’s work-life balance along with the culture amongst the fellows themselves. The use of social media and virtual technologies has been shown to markedly increase program visibility [23]. Czawlytko et al. discussed their program’s use of virtual open houses to attract applicants and found that 69% of their applicants reported attending a virtual open house and 57% of those attending stated that this influenced their decision to apply [21]. Similarly, maximizing website content and functionality can considerably influence applicants’ program interview selection and ranking [24-26]. Through these tools, a program can distinguish itself from others, show the facilities, and share organic interactions between fellows, faculty, and the multidisciplinary team, to highlight the program culture.

Other gaps in the VI day included the lack of virtual tours or program videos. As in-person interviews typically included shadowing rounds and attending lectures or hospital tours, incorporating a sense of daily activities and the working environment should be considered in organizing the VI. Aligning with this, many survey respondents reported wishing they had virtual tours or program videos included in their VI. Additionally, respondents reported wanting to meet with ancillary personnel such as APPs or nursing leadership, as these relationships significantly shape their daily clinical environment. In cases where meeting with ancillary personnel may not be possible, including them in a video or virtual tour may be beneficial for understanding the hospital community and relationships.

This study had several limitations. The low number of responses may limit the generalizability of these results as the respondent group may not be representative of all NPM fellows or programs. Respondents were removed from the analysis if they previously worked or trained at their institution, which further limited the study population. Compared to the demographic data of the USA and Canada NPM fellows in recent years, we saw a greater percentage of male respondents and Doctorate of Medicine (MD) training credentials, which potentially skews the representation of programs [27]. However, all regions of the USA and Canada were represented demographically amongst the respondents, which strengthens the breadth of representation. Within the survey, respondents were not asked about the position of their respective program on their rank list, which may have influenced their opinion about the program. The survey structure and timing required respondents to comment on the VI day and their perception of program culture in the year following the VI, potentially leading to recall bias. Additionally, as a multitude of factors likely impact trainee perceptions of program culture, we cannot make direct conclusions about program culture as represented in the VI.

## Conclusions

This novel study adds to the VI literature and confirms the inherent challenge of representing program culture through a virtual format. Considering the many advantages of VIs, it is important to examine approaches to enhance VIs and promote a clearer understanding of program culture. Potential strategies include increased fellow involvement, implementing more casual settings such as happy hours, the usage of social media, and illustrating the day-to-day interactions of a trainee, such as through virtual tours or meetings with other members of the multidisciplinary team. While virtual encounters may never fully replicate in-person interactions, it is possible that addressing these gaps may improve the interview process for both applicants and programs.

## Appendices

**Table 2:** Survey of first-year neonatology fellows' perceptions of program culture through virtual interviews during COVID-19

Question	Answer choices/Free text
DATE	
1 Prior to fellowship, did you ever work or train (medical school, residency) at the institution where you are currently completing fellowship?	Yes, please exit the survey No
2 Did you complete a virtual interview at the institution where you matched for fellowship?	Yes No, please exit the survey
The following questions relate to your virtual interview experience of the program at which you matched for fellowship.	
3 What was the approximate duration of the interview day?	2-4 hours 5-6 hours 7-8 hours > 8 hours
4 What activities were included in your virtual interview day? (Select all that apply)	Outline of the program by Program Director/Associate Program Director (either informally, via PowerPoint, or another platform) Individual interview with the Program Director/Associate Program Director Individual interviews with faculty members within the Division of Neonatology Individual interviews with faculty members outside the Division of Neonatology (e.g., pediatric cardiology, neurology, etc.) Interviews/meeting with the current fellows Interviews/meeting with Advanced Practice Providers (i.e., nurse practitioners, physician assistants) Interviews/meeting with NICU nursing leadership (i.e., unit nursing director, etc.) Interviews/meeting with allied health staff (e.g., pharmacist, respiratory therapist, etc.) Meeting with the Program Coordinator Applicant group activity (e.g., role-play, problem-solving activity, etc.) Program video Hospital and/or NICU virtual tour Other (describe and be specific) ______________________
5 Did the program offer other meetings with the current fellows SEPARATE from the interview day?	Yes (select all that apply) No Select all that apply. i. Virtual happy hour ii. Virtual lunch/dinner iii. Virtual café iv. Other (describe and be specific) ______________________
6 Did the program use social media to provide additional information regarding the activities, upcoming events etc.?	Yes No Describe ______________________
The following questions relate to your perception of your fellowship program's culture BASED ON YOUR VIRTUAL INTERVIEW EXPERIENCE (i.e., before you started your fellowship training). 1=Strongly Disagree, 5 = Strongly Agree	
7 There appeared to be a positive and respectful relationship between the FELLOWS.	Strongly disagree Disagree Neither agree or disagree Agree Strongly agree Not applicable
8 There appeared to be a positive and respectful relationship between the FACULTY MEMBERS.	Strongly disagree Disagree Neither agree or disagree Agree Strongly agree Not applicable
9 There appeared to be a positive and respectful relationship between the FACULTY MEMBERS and the FELLOWS.	Strongly disagree Disagree Neither agree or disagree Agree Strongly agree Not applicable
10 The program and faculty appeared to place a high priority on fellow teaching.	Strongly disagree Disagree Neither agree or disagree Agree Strongly agree Not applicable
11 The program appeared to respect and value the fellows.	Strongly disagree Disagree Neither agree or disagree Agree Strongly agree Not applicable
12 The program appeared to value fellow work-life integration.	Strongly disagree Disagree Neither agree or disagree Agree Strongly agree Not applicable
13 Overall, the fellows' attitudes toward the applicants on interview day were	Very positive Somewhat positive Neutral Somewhat negative Very negative
14 Overall, the faculty's attitudes toward the applicants on interview day were	Very positive Somewhat positive Neutral Somewhat negative Very negative
15 Please indicate who/what contributed most significantly to your impression of the culture of the program during your virtual interview day (Please be specific).	
16 What do you wish your program's virtual interview day had included that would have contributed to your impression of the culture of the program? (Please be specific).	
The following questions relate to your perception of your fellowship program's culture BASED ON YOUR ACTUAL EXPERIENCE (i.e., after you started your fellowship training). 1=Strongly Disagree, 5=Strongly Agree	
17 There is a positive and respectful relationship between the FELLOWS.	Strongly disagree Disagree Neither agree or disagree Agree Strongly agree Not applicable
18 There is a positive and respectful relationship between the FACULTY MEMBERS.	Strongly disagree Disagree Neither agree or disagree Agree Strongly agree Not applicable
19 There is a positive and respectful relationship between the FACULTY MEMBERS and the FELLOWS.	Strongly disagree Disagree Neither agree or disagree Agree Strongly agree Not applicable
20 The program and faculty place a high priority on fellow teaching.	Strongly disagree Disagree Neither agree or disagree Agree Strongly agree Not applicable
21 The program respects and values the fellows.	Strongly disagree Disagree Neither agree or disagree Agree Strongly agree Not applicable
22 The program values fellow work-life integration.	Strongly disagree Disagree Neither agree or disagree Agree Strongly agree Not applicable
23 Please rate how well your perception of your program's culture - as portrayed through virtual interviews - aligns with your direct experience of your program's culture.	0% ----------------------------------------------- 100% (Place a mark on the scale above)
Demographics	
23 What is your age?	25-30 31-35 36-40 >40
24 What is your gender?	Female Male Prefer not to say Other
25 What are your credentials?	MD - US medical school MD - non-US medical school DO - US medical school DO - non-US medical school MBBS Other (Specify) ______________________
26 In which country are you in fellowship training?	USA Please select region i. Northeast ii. Southeast iii. Midwest iv. Southwest v. West Canada Province/Territory: _____________________
27 What is your PRIMARY program type?	University hospital Community hospital, university affiliated Community hospital, non-university affiliated Other (Describe) ______________________
28 Approximately how fellowship programs did you interview with?	1-5 6-10 11-20 >20
29 Questions/Comments	
